# Mathematics teaching outcome expectancy, self-handicapping, and mathematics teaching anxiety: a two-wave mediation study

**DOI:** 10.3389/fpsyg.2026.1887406

**Published:** 2026-07-29

**Authors:** Muhammed Celal Uras

**Affiliations:** Department of Mathematics Education, Aǧrı İbrahim Çeçen University, Aǧrı, Türkiye

**Keywords:** mathematics teaching anxiety, mathematics teaching outcome expectancy, preservice classroom teachers, self-handicapping, two-wave mediation

## Abstract

**Background:**

Beliefs about the instructional impact of teaching may shape how preservice classroom teachers respond to demanding mathematics teaching. Mathematics teaching anxiety and self-handicapping also play an important role. The present study tested a two-wave mediation model examining the mediating roles of anxiety related to teaching and anxiety related to subject knowledge in the relationship between mathematics teaching outcome expectancy and self-handicapping.

**Methods:**

A total of 180 Turkish preservice classroom teachers (130 female), aged 18–27 (*M*_age_ = 21.78, SD = 1.96), participated in this study. Participants completed an online survey including the Teaching Mathematics Efficacy Belief Instrument, Mathematics Teaching Anxiety Scale, and Self-Handicapping Scale.

**Results:**

The findings showed that mathematics teaching outcome expectancy was negatively associated with self-handicapping, anxiety related to teaching, and anxiety related to subject knowledge. The results also revealed that anxiety related to teaching and anxiety related to subject knowledge had parallel mediating effects in the relationship between mathematics teaching outcome expectancy and self-handicapping.

**Conclusions:**

The findings suggest that stronger beliefs about the effects of teaching may reduce self-protective coping among preservice classroom teachers, partly by lowering mathematics teaching anxiety. These results are important because they indicate that strengthening mathematics teaching outcome expectancy and addressing different dimensions of mathematics teaching anxiety may help reduce self-handicapping during teacher training.

## 1 Introduction

Mathematics learning in the early years has a lasting role in children's later academic development. Early numeracy is widely regarded as a foundational component of mathematics achievement. Longitudinal evidence shows that growth in early number competence predicts performance in primary school mathematics (Dierkx et al., 2025; Jordan et al., 2022). Early mathematics skills also have consequences beyond mathematics alone. ten Braak et al. (2022) reported that early mathematics predicts reading achievement as well, with executive function helping to explain part of that association. Therefore, literature places early mathematics learning near the center of children's broader academic development. The instructional significance of this period makes classroom teachers central to children's mathematical development. In primary grades, teachers introduce foundational ideas about number, operations, and representation while also shaping students' early learning experiences. Therefore, teacher preparation involves more than acquiring mathematical content knowledge (Ball et al., 2008). It also involves the development of professional beliefs regarding whether instruction will influence student learning and how to effectively manage pedagogical challenges. Research with preservice teachers has demonstrated that mathematics teaching efficacy beliefs, including beliefs about the instructional impact of teaching, develop during teacher education and are shaped by mathematics-related experiences and perceived preparedness (Bandura, 1997; Power et al., 2026; Twohill et al., 2023). Studies with Turkish preservice teachers also indicate that self-efficacy beliefs toward mathematics teaching are salient during undergraduate preparation (Bursal, 2010; Çakiroglu and Işiksal, 2009).

For preservice classroom teachers, mathematics often becomes a domain in which competence is publicly tested. Explaining ideas clearly, selecting suitable representations, managing students' responses, and dealing with unexpected errors can all make mathematics teaching emotionally demanding. In such situations, weaker beliefs about the likely effects of teaching may be accompanied by stronger teaching-related anxiety, especially when instructional demands are experienced with limited perceived control (Pekrun, 2024; Peker, 2009). It may also increase the appeal of defensive responses. When challenging teaching tasks are experienced as threats to competence, preservice teachers may resort to self-protective responses such as avoidance, reduced effort, or self-handicapping. This pattern has been observed in preservice teachers facing self-worth threats and is consistent with broader evidence defining self-handicapping as a strategy of effort reduction and obstacle creation to protect self-image (Barkela et al., 2024). Within this context, mathematics teaching outcome expectancy, mathematics teaching anxiety, and self-handicapping can be understood as closely connected aspects of professional preparation. The present study focuses on preservice classroom teachers because they are preparing for a role that is especially consequential in the early years of schooling. Professional beliefs about teaching are still taking shape, and mathematics often becomes a domain in which competence, uncertainty, and emotional reactions are experienced quite visibly. A model that links mathematics teaching outcome expectancy, mathematics teaching anxiety, and self-handicapping is relevant both to teacher education and to the quality of future classroom instruction.

### 1.1 Literature review

#### 1.1.1 Mathematics teaching outcome expectancy in preservice classroom teachers

Within social cognitive theory, efficacy beliefs refer to judgments about one's capability to organize and carry out actions required to attain desired outcomes (Bandura, 1997). In mathematics teacher education, these beliefs have been operationalized in a field-specific manner by Enochs et al. (2000). They distinguish between personal mathematics teaching efficacy and expectations regarding the outcomes of mathematics instruction. Mathematics teaching outcome expectancy refers to the belief that effective teaching can improve students' mathematics learning outcomes, even when external factors may also matter (Enochs et al., 2000). Moreover, the construct concerns beliefs about the instructional power of teaching rather than liking for mathematics or a broad positive attitude toward the profession (Hourigan and Leavy, 2022; Twohill et al., 2023). It has particular relevance for classroom teacher education. Primary teachers are expected to teach mathematics in ways that make abstract ideas understandable to young learners. It requires explanation, representation, questioning, monitoring, and instructional adjustment. Preservice teachers who believe that teaching can genuinely influence learning may be more likely to persist when students struggle. Research has revealed that mathematics teaching efficacy beliefs are related to preparedness to teach, prior mathematics attainment, and features of teacher education programs (Işiksal-Bostan, 2016; Charitaki et al., 2026). Evidence from Turkey also indicates that preservice teachers' mathematics-related self-efficacy beliefs are professional resources during teacher preparation (Bursal, 2010; Çakiroglu and Işiksal, 2009).

Expectations regarding outcomes do not merely reflect personal self-confidence. A preservice teacher may lack confidence in their current teaching skills, yet still believe that effective teaching can support children's learning. On the other hand, while personal self-confidence may be relatively strong, beliefs about the actual impact of teaching may remain weak. This distinction has been emphasized in studies on mathematics teaching effectiveness, where outcome expectations and personal efficacy are treated as interrelated yet distinct dimensions (Enochs et al., 2000; Twohill et al., 2023). For preservice teachers, low expectations regarding outcomes can make the challenges of teaching seem even more unmanageable. Furthermore, in situations where mathematics instruction is perceived as evaluative or threatening, this can increase susceptibility to anxiety and defensive coping strategies.

In the present study, mathematics teaching outcome expectancy is not treated as an isolated professional belief. It is considered in relation to both mathematics teaching anxiety and self-handicapping. When preservice classroom teachers are less convinced that effective teaching can improve student learning, instructional difficulty may be experienced with a weaker sense of control. In mathematics teaching, this may increase sensitivity to evaluative situations and strengthen anxiety related to both the teaching and subject knowledge. Previous studies using broader mathematics teaching efficacy frameworks have shown that beliefs about mathematics teaching are connected with anxiety-related experiences during teacher preparation and may continue to develop across preparation and early professional practice (Gresham, 2008; Swars et al., 2006; Işiksal-Bostan, 2016). However, the present study focuses specifically on the outcome expectancy dimension rather than personal mathematics teaching efficacy. From this perspective, weaker expectations about the instructional impact of teaching may be associated with greater anxiety because preservice teachers may feel that their instructional effort has limited influence on students' learning. Evidence also suggests that weaker professional beliefs may be linked with stronger self-protective tendencies in student and preservice teacher populations, including self-handicapping (Akgöz et al., 2025; Goodarznaseri et al., 2026). Thus, mathematics teaching outcome expectancy is treated here as an early belief about instructional impact that helps explain later emotional and behavioral responses in mathematics teacher preparation.

#### 1.1.2 Mathematics teaching anxiety in preservice teachers

Mathematics teaching anxiety is closely tied to professional demands. Anxiety is not limited to general uneasiness about mathematics content. It also involves the stress associated with explaining mathematical concepts, managing the flow of the lesson, selecting appropriate methods, and answering students' questions during instruction (Peker, 2009). Research has examined anxiety about teaching mathematics as a meaningful concept for preservice elementary school teachers and has investigated it through the development of specialized scales and validation studies (Alkan et al., 2019). Control-value theory proposes that anxiety tends to emerge when achievement situations are appraised as highly valuable but low in perceived control (Pekrun, 2006). Mathematics teaching is consistent with that description for many preservice classroom teachers because pedagogical performance and subject knowledge are simultaneously exposed to evaluation (Abuan and Taganap, 2025).

Anxiety and beliefs about teaching mathematics have frequently been examined together in research on preservice teachers (Gresham, 2008; Uysal and Dede, 2016). Swars et al. (2006) found a moderate negative relationship between mathematics anxiety and mathematics teacher efficacy among elementary preservice teachers. Although such studies often use broader mathematics teaching efficacy measures, they provide relevant background for the present study because outcome expectancy is one theoretically distinct component of the mathematics teaching efficacy beliefs framework. Mizala et al. (2015) showed that preservice elementary teachers' mathematics anxiety shapes expectations about student performance. It gives anxiety a direct place in classroom judgment rather than treating it as a purely internal emotional condition. Aksu and Kul (2019) reported that mathematics teaching efficacy plays a mediating role in the relationship between pedagogical content knowledge and mathematics teaching anxiety. Evidence also indicates that mathematics anxiety can mediate the association between technology-related teaching competencies and mathematics teaching anxiety in preservice teachers (Çetin and Yazlik, 2022).

Two dimensions of anxiety are especially relevant here. One concerns anxiety related to teaching, including explanation, classroom interaction, lesson management, and assessment. The other concerns anxiety related to subject knowledge, especially the fear of not knowing enough mathematics to teach flexibly and convincingly. Anxiety related to subject knowledge deserves separate theoretical attention. It reflects concerns about mathematical adequacy during instruction. Preservice classroom teachers are expected to explain foundational mathematical ideas. They must respond to students' unexpected questions, correct errors, and select representations that make concepts understandable (Ball et al., 2008; Copur-Gencturk and Tolar, 2022). These tasks may expose perceived gaps in mathematical understanding. This can happen even when the preservice teacher is generally motivated to teach. Therefore, anxiety related to subject knowledge differs from anxiety related to teaching. The former concerns whether one's mathematical understanding is sufficient for instruction. The latter concerns lesson enactment, classroom interaction, and instructional management. A preservice teacher may feel anxious about managing the teaching process, about the mathematical content itself, or about both. Research from Turkey has repeatedly shown that mathematics teaching anxiety is meaningfully connected with beliefs about teaching and learning mathematics among preservice primary school teachers (Başpinar and Peker, 2016; Peker, 2009). Studies conducted with elementary school teachers noted that mathematics teaching anxiety and mathematics teaching efficacy are systematically related (Göçer and Özeren, 2025). Therefore, distinguishing anxiety related to teaching from anxiety related to subject knowledge may clarify how professional beliefs are linked with later maladaptive responses in teacher preparation.

In the context of the present study, mathematics teaching anxiety is important because it may represent the mechanism through which earlier outcome expectancy beliefs are linked with later self-handicapping. Lower outcome expectancy may reduce the perceived instructional influence of teaching, and reduced perceived control may intensify anxiety in situations where mathematics teaching is publicly evaluated. That process is theoretically consistent with control-value theory (Pekrun, 2024) and also aligns with empirical work showing that mathematics-related anxiety is negatively associated with efficacy-related beliefs in preservice teachers (Gresham, 2008; Başpinar and Peker, 2016). The same pattern has also been observed in more recent studies, where stronger anxiety has been connected with weaker beliefs about mathematics teaching, including beliefs about the instructional effectiveness of teaching (Abuan and Taganap, 2025; Lavidas et al., 2023). In the present study, anxiety related to teaching and anxiety related to subject knowledge are positioned as emotional variables. They are also treated as two distinct pathways linking professional beliefs with later defensive coping.

#### 1.1.3 Self-handicapping

Self-handicapping refers to the creation or claim of obstacles before performance so that possible failure can be attributed to obstacles (Urdan and Midgley, 2001). Within the framework of self-worth theory, such defensive behaviors are viewed as part of a broader effort to protect one's self-worth under evaluative conditions (Covington, 1984). Academic settings are especially fertile ground for this process because competence is repeatedly tested and failure can easily be interpreted as evidence of low ability. Delay, excuse making, reduced effort, and avoidance of demanding tasks are among the best-known forms of self-handicapping (Urdan and Midgley, 2001). These features make self-handicapping especially relevant in teacher education, where competence is repeatedly displayed, interpreted, and judged in instructional settings. Teacher education includes many of the conditions under which self-protective behavior becomes likely. Preservice teachers are repeatedly asked to display knowledge, pedagogical judgment, and classroom readiness in settings where performance is observed and evaluated. Research on preservice teachers has shown that self-worth threat can increase resistance to engagement and promote self-protective behavior when professional learning is experienced as threatening (Barkela et al., 2024). Evidence from preservice teacher samples has shown that self-handicapping tendencies are observable during teacher preparation, and this has led researchers to emphasize the need for coping-oriented support in teacher education programs (Sofo et al., 2025). Research has also documented self-handicapping strategies such as procrastination and avoidance in academic learning contexts (Ganda and Boruchovitch, 2015; Gündogdu, 2013; Kaya et al., 2017). In teacher training, defensive behavior may take the form of underpreparing for teaching tasks, withdrawing from difficult instructional opportunities, or limiting engagement in feedback-rich learning situations. For preservice classroom teachers, the relevance of self-handicapping is heightened by the character of mathematics teaching itself. Mathematics lessons often demand public explanation, rapid response to errors, and visible demonstrations of understanding. When preservice teachers doubt the impact of instruction and simultaneously feel anxious about teaching mathematics, self-protective strategies may become more appealing. Therefore, a model that links outcome expectancy, mathematics teaching anxiety, and self-handicapping addresses a meaningful process in professional preparation rather than a psychological association.

The concept of self-handicapping was considered as a behavioral outcome in the present study. In teacher education, defensive responses may become more likely when demanding instructional tasks are experienced as threatening to competence. Preservice classroom teachers who hold weaker beliefs about the likely impact of teaching may be more vulnerable to this risk, particularly when mathematics teaching also evokes anxiety about lesson delivery or subject knowledge. Research has shown that lower efficacy beliefs are associated with stronger self-handicapping tendencies in student samples, and related work with preservice teachers points in a similar direction (Akgöz et al., 2025; Goodarznaseri et al., 2026; Gündogdu, 2013). This makes self-handicapping more than a general academic tendency in the present study. It is treated as a self-protective response that may emerge when lower outcome expectancy and stronger mathematics teaching anxiety combine during a formative stage of professional preparation.

### 1.2 The present study

Three complementary perspectives frame the present study. Social cognitive theory distinguishes between beliefs about personal capability and expectations about the outcomes of action, and the present study focuses on the latter in the context of mathematics teaching (Bandura, 1997). Control-value theory explains why anxiety emerges when valued activities are experienced as difficult to control (Pekrun, 2024). Self-worth theory clarifies why threat to competence may trigger defensive behavior designed to protect personal value (Covington, 1984; Urdan and Midgley, 2001). These perspectives support a coherent interpretation of the process examined. Weaker mathematics teaching outcome expectancy may reduce the sense that instructional effort can influence learning. Lower perceived instructional control may then intensify anxiety during mathematics teaching. Outcome expectancy matters in mathematics teaching because it reflects whether teaching is believed to influence student learning. Weaker outcome expectancy can make teaching tasks feel less controllable, which, from a control-value perspective, intensifies anxiety around important or evaluative tasks. This anxiety may center on the teaching process itself or on whether one's subject knowledge is sufficient to handle students' questions and errors. Thus, weaker outcome expectancy may be linked with both forms of anxiety through reduced perceived control, consistent with broader teacher education research on self-efficacy, self-directed learning, and classroom-management anxiety, and with recent mediation-based studies of preservice mathematics teachers (Gürbüz et al., 2026; Özkul, 2025; Özkul and Dönmez, 2023; Uras et al., 2026).

Empirical research supports the same pathway. Prior studies using mathematics teaching efficacy frameworks have linked preservice teachers' beliefs about mathematics teaching with preparedness to teach and teacher education experiences (Hourigan and Leavy, 2022; Power et al., 2026; Twohill et al., 2023). In the present study, this broader literature is used to situate mathematics teaching outcome expectancy, not to treat outcome expectancy and personal teaching efficacy as interchangeable constructs. A negative association between mathematics-related anxiety and mathematics teaching efficacy has been documented across preservice teacher studies (Gresham, 2008; Swars et al., 2006). Research in Türkiye has also shown that mathematics teaching anxiety is related to beliefs about teaching and learning mathematics and to broader professional competencies (Başpinar and Peker, 2016; Çetin and Yazlik, 2022; Uysal and Dede, 2016). Studies on the threat to self-worth and self-protective behavior in teacher education also suggest that expectancy-related beliefs and anxiety may feed into defensive behavioral tendencies (Barkela et al., 2024). Nevertheless, longitudinal evidence remains limited, outcome expectancy is often absorbed into broader efficacy models, and self-handicapping has rarely been studied together with distinct dimensions of mathematics teaching anxiety in preservice classroom teachers. The present study was designed to address that gap. Mathematics teaching outcome expectancy measured at Time 1 was specified as the predictor. Anxiety related to teaching at Time 2 and anxiety related to subject knowledge at Time 2 were specified as parallel mediators. Self-handicapping at Time 2 was specified as the dependent variable. Because all variables were measured at both time points, the two-wave design also allowed the study to examine rank-order stability and mean-level change across the 6-month interval. Mathematics teaching outcome expectancy, mathematics teaching anxiety, and self-handicapping may show both continuity and change during teacher education. Accordingly, before testing the mediation model, the study examined whether participants maintained their relative standing on each construct from Time 1 to Time 2 and whether the group-level means of the constructs changed across waves. Time 1 mathematics teaching outcome expectancy was retained as the predictor because the structural model focused on whether earlier beliefs about the instructional impact of teaching were associated with later anxiety and self-handicapping, rather than on concurrent associations among constructs at Time 2. Examining these pathways in preservice classroom teachers may clarify whether early professional beliefs about the instructional power of teaching are linked with later defensive academic behavior through two forms of mathematics teaching anxiety that are highly relevant to primary instruction.

The hypotheses were as follows:

H1: Mathematics teaching outcome expectancy at Time 1 is negatively associated with self-handicapping at Time 2.

H2: The relationship between mathematics teaching outcome expectancy at Time 1 and self-handicapping at Time 2 is mediated by anxiety related to teaching at Time 2.

H3: The relationship between mathematics teaching outcome expectancy at Time 1 and self-handicapping at Time 2 is mediated by anxiety related to subject knowledge at Time 2.

H4: The relationship between mathematics teaching outcome expectancy at Time 1 and self-handicapping at Time 2 is mediated jointly by anxiety related to teaching and anxiety related to subject knowledge at Time 2.

## 2 Method

### 2.1 Participants

The study used a two-wave design with a 6-month interval between assessments. At Time 1, the initial sample consisted of 195 participants. Data were collected again 6 months later at Time 2. After the matching procedure was completed, the final sample included 180 preservice classroom teachers. Of these participants, 130 were female (72%), and 50 were male (28%). Their ages ranged from 18 to 27 years, with a mean age of 21.78 years (SD = 1.96). The sample comprised 25 first-year students (13.9%), 40 second-year students (22.2%), 67 third-year students (37.2%), and 48 fourth-year students (26.7%). All participants were enrolled in a 4-year classroom teacher education program at a public university in Türkiye. This program prepares preservice classroom teachers to teach core subjects, including mathematics, at the primary-school level. Depending on their year of study, participants had completed or were completing coursework related to primary mathematics teaching, instructional methods, and teaching practice. Thus, the sample represented preservice classroom teachers at different stages of professional preparation for teaching mathematics in the primary grades.

### 2.2 Procedure

All stages of the research were conducted in accordance with the 1975 Declaration of Helsinki and its subsequent revisions. Data were collected through convenience sampling by means of an online form prepared in Google Forms. The form explained the longitudinal structure of the study, provided an estimate of the completion time, and included the necessary information about the research. Participants gave informed consent before completing the form. They were also informed that participation was voluntary and that they could withdraw from the study at any time. Participants were eligible if they were at least 18 years old, enrolled in a classroom teacher education program at a public university, and agreed to participate voluntarily. No monetary compensation or other incentive was offered. To protect confidentiality and ensure accurate matching across waves, participants were instructed not to provide personally identifying information. Instead, they created a code using the last five digits of their student number and the last five digits of their phone number. This code was used to match responses over time. Multiple submissions were not permitted.

### 2.3 Measures

All study variables were measured at both Time 1 and Time 2. However, in the structural model, mathematics teaching outcome expectancy measured at Time 1 was specified as the predictor, whereas anxiety related to teaching, anxiety related to subject knowledge, and self-handicapping measured at Time 2 were specified as the mediators and dependent variables, respectively. Measuring all variables at both waves served three purposes. First, it allowed the internal consistency of each instrument to be evaluated separately at Time 1 and Time 2. Second, it enabled the examination of rank-order stability and mean-level change across the 6-month interval. Third, it provided a descriptive account of how the constructs behaved over time, even though the structural model focused on a theory-driven pathway from Time 1 mathematics teaching outcome expectancy to Time 2 anxiety and self-handicapping.

#### 2.3.1 Mathematics teaching outcome expectancy

The subscale of mathematics teaching outcome expectancy from the Teaching Mathematics Efficacy Belief Instrument by Enochs et al. (2000) [Turkish version: (Takunyaci, 2021)] was used to assess outcome expectancy. The subscale consists of 8 items and is designed to assess preservice teachers' beliefs about the extent to which effective teaching can influence students' mathematics learning outcomes. Sample items include “The inadequacy of a student's mathematics background can be overcome by good teaching.” Items are rated on a 5-point Likert scale ranging from 1 (Strongly Disagree) to 5 (Strongly Agree). The overall score ranges from 8 to 40. Higher scores indicate stronger mathematics teaching outcome expectancy, that is, stronger beliefs that effective mathematics teaching can improve students' mathematics learning. The scale showed goodness-of-fit indices for a one-factor model at Time 1 (χ^2^ = 28.272, df = 20, χ^2^/df = 1.41, CFI = 0.95, IFI = 0.95, TLI = 0.93, GFI = 0.99, SRMR = 0.06 and RMSEA = 0.05). In the present study, Cronbach's α and McDonald's ω were 0.75 and 0.75 at Time 1 and 0.86 and 0.86 at Time 2, respectively.

#### 2.3.2 Mathematics teaching anxiety

Mathematics teaching anxiety was assessed using three relevant subscales of the 23-item Mathematics Teaching Anxiety Scale, originally developed by Sarı (2014) to measure anxiety levels of classroom teachers while teaching mathematics. In the present study, the anxiety related to teaching and anxiety related to subject knowledge subscales were used.

The anxiety related to teaching subscale consists of 11 items and measures anxiety associated with instructional processes in mathematics teaching. A sample item, translated from Turkish, is “The thought that the class may not be successful in mathematics lessons worries me.” Items are rated on a five-point Likert scale ranging from 1 (never) to 5 (always). Total scores range from 11 to 55. The scale showed goodness-of-fit indices for a one-factor model at Time 2 (χ^2^ = 74.071, df = 44, χ^2^/df = 1.68, CFI = 0.91, IFI = 0.92, GFI = 0.99, SRMR = 0.06, and RMSEA = 0.06). In the present study, Cronbach's α and McDonald's ω were 0.93 and 0.93 at Time 1 and 0.93 and 0.93 at Time 2, respectively.

The anxiety related to the subject knowledge subscale consists of 6 items and captures anxiety stemming from perceived inadequacies in mathematics subject knowledge during teaching. A sample item, translated from Turkish, is “Knowing that the next lesson is mathematics makes me uneasy.” Items are also rated on a five-point Likert scale ranging from 1 (never) to 5 (always). Total scores range from 6 to 30. In the present study, the scale showed goodness-of-fit indices for a one-factor model at Time 2 (χ^2^ = 13.208, df = 9, χ^2^/df = 1.47, CFI = 0.98, IFI = 0.99, GFI = 0.99, SRMR = 0.03, and RMSEA = 0.05). In the present study, Cronbach's α and McDonald's ω were 0.91 and 0.91 at Time 1 and 0.90 and 0.90 at Time 2, respectively.

#### 2.3.3 Self-handicapping

Self-handicapping was assessed using the 10-item short form of the Self-Handicapping Scale, originally developed by Jones and Rhodewalt (1982) [Short form: Strube (1986); Turkish version: Akin (2012)]. The scale assesses undergraduate students' tendency to engage in self-handicapping behaviors. Example items include “I tend to make excuses when I do something wrong.” Items are rated on a six-point Likert scale ranging from 1 (strongly disagree) to 6 (strongly agree). The total score ranges from 10 to 60, with higher scores indicating higher levels of self-handicapping. In the present study, the one-factor model showed acceptable fit at Time 2 (χ^2^ = 68.875, df = 35, χ^2^/df = 1.97, GFI = 0.98, SRMR = 0.08, and RMSEA = 0.07). In the present study, Cronbach's α and McDonald's ω were 0.62 and 0.63 at Time 1 and 0.69 and 0.72 at Time 2, respectively.

### 2.4 Data analysis

Data analyses were performed using SPSS 26, AMOS Graphics 24, and JASP 0.96. Before testing the main model, the distributional properties of the data were evaluated through skewness and kurtosis values. Values within acceptable limits were taken as evidence that the variables did not depart substantially from normality (Kline, 2023). Multicollinearity was then examined by inspecting tolerance, variance inflation factor (VIF), and condition index values. Tolerance values above 0.20, VIF values below 10, and condition index values below 30 were treated as indicating that multicollinearity was not a serious concern. All values fell within these ranges. Therefore, the data were considered suitable for the subsequent analyses.

Before testing the mediation model, the two-wave structure of the data was examined descriptively. Rank-order stability was assessed using Pearson correlations between Time 1 and Time 2 scores for each variable. Mean-level change was examined using paired-samples *t*-tests. Cohen's d values were calculated to evaluate the magnitude of mean-level change. These analyses were conducted to clarify the degree to which the constructs showed continuity or change across the 6-month interval. After the assumptions were checked, the hypotheses were tested by using a two-step structural equation modeling procedure. In line with Anderson and Gerbing (1988), the measurement model was evaluated first, and the structural model was tested afterward. Model fit was assessed with the comparative fit index (CFI), incremental fit index (IFI), goodness of fit index (GFI), standardized root mean square residual (SRMR), and root mean square error of approximation (RMSEA). Consistent with commonly used reporting guidelines, CFI, IFI, and GFI values of 0.90 or above and RMSEA and SRMR values below 0.08 were taken to indicate acceptable fit (Schreiber et al., 2006). Because the structural model included several latent variables and the sample size was relatively modest, item parceling was used to obtain a more parsimonious measurement structure and to reduce the number of estimated parameters. Parcels were created separately for each construct after the one-dimensionality of the relevant subscales had been confirmed. Following an item-to-construct balance approach, items were distributed across parcels to achieve relatively balanced construct representation. The item-to-construct balance strategy was used to avoid placing items with similar psychometric strength into the same parcel. After the unidimensional structure of each subscale was supported, items were ordered according to their corrected item-total correlations and then distributed across parcels so that each parcel represented the construct as evenly as possible. Parcel scores were computed as the mean of the items assigned to each parcel. This strategy was selected to reduce the number of estimated parameters, improve model parsimony, and maintain stable estimation given the sample size. Two parcels were formed for mathematics teaching outcome expectancy, two for anxiety related to teaching, two for anxiety related to subject knowledge, and three for self-handicapping. Parcel scores were calculated by averaging the items assigned to each parcel. Age and gender were also entered into the structural model as covariates in order to control for possible demographic differences in the endogenous variables. Within this analytic framework, the model tested whether mathematics teaching outcome expectancy at Time 1 predicted self-handicapping at Time 2 through the parallel mediating roles of anxiety related to teaching and anxiety related to subject knowledge at Time 2. Time 2 mathematics teaching outcome expectancy was not specified as the main predictor in the structural model because doing so would have shifted the analysis from a two-wave mediation model to a concurrent Time 2 model. The structural model aimed to test a theory-driven pathway from earlier outcome expectancy to later anxiety and self-handicapping. However, correlations involving Time 2 mathematics teaching outcome expectancy were reported and discussed to avoid overlooking its concurrent pattern of associations.

#### 2.4.1 Attrition analysis

195 participants who completed at T1, 180 (92.3%) completed at T2, resulting in an attrition rate of 7.7% (*n* = 15). To assess potential attrition bias, we compared completers (*n* = 180) and non-completers (*n* = 15) on baseline demographics (age and sex) and baseline study variables using independent-samples *t*-tests and chi-square tests. Results indicated no evidence of selective attrition. Completers and non-completers did not differ in age [M = 21.78, SD = 1.96 vs. M = 21.07, SD = 1.28; *t*_(193)_ = 1.37, *p* = 0.172]. Sex distribution also did not differ significantly between groups [χ^2^(1, *N* = 195) = 3.195, *p* = 0.074]. Similarly, completers and non-completers did not differ significantly on baseline mathematics teaching outcome expectancy [*t*_(193)_ = −0.194, *p* = 0.846], self-handicapping [*t*_(193)_ = −0.201, *p* = 0.841], anxiety related to teaching (*t*_(193)_ = −0.154, *p* = 0.878), or anxiety related to subject knowledge [*t*_(193)_ = 0.897, *p* = 0.371]. Overall, these findings suggest that attrition between T1 and T2 is unlikely to have introduced substantial bias into longitudinal analyses.

## 3 Results

### 3.1 Preliminary analysis

Table 1 presents the descriptive statistics, correlation coefficients, and reliability estimates for the study variables, including means, standard deviations, skewness, and kurtosis values. The Pearson correlation analysis indicated that mathematics teaching outcome expectancy, anxiety related to teaching, anxiety related to subject knowledge, and self-handicapping were significantly associated with one another at weak to moderate levels. Both positive and negative correlations were observed across the variables. Mathematics teaching outcome expectancy was negatively correlated with self-handicapping at Time 2 (*r* = −0.39, *p* < 0.01). It was also negatively associated with anxiety related to teaching (*r* = −0.17 and *r* = −0.17, within the respective ranges, *p* < 0.05) and anxiety related to subject knowledge (*r* = −0.26 and *r* = −0.21, within the respective ranges, *p* < 0.01). Self-handicapping, in contrast, was positively associated with anxiety related to teaching at both time points (*r* = 0.45 and *r* = 0.31, within the respective ranges, *p* < 0.01) and with anxiety related to subject knowledge at both time points (*r* = 0.34 and *r* = 0.28, within the respective ranges, *p* < 0.01). Reliability analyses were conducted separately for Time 1 and Time 2. Cronbach's α and McDonald's ω values are reported in Table 1. Across the two measurement points, the internal consistency estimates ranged from 0.62 to 0.94, indicating acceptable to high reliability for most measures. The self-handicapping scale showed comparatively lower reliability and is therefore interpreted with caution.

**Table 1:** Descriptive statistics and reliabilities for the study variables.

Variable	1	2	3	4	5	6	7	8
1. Mathematics teaching outcome expectancy T1	–							
2. Mathematics teaching outcome expectancy T2	0.29^**^	–						
3. Anxiety related to teaching T1	−0.17^*^	−0.07	–					
4. Anxiety related to teaching T2	−0.17^*^	0.06	0.30^**^	–				
5. Anxiety related to subject knowledge T1	−0.26^**^	−0.18^*^	0.22^**^	0.07	–			
6. Anxiety related to subject knowledge T2	−0.21^**^	−0.16^*^	0.16^*^	0.27^**^	0.23^**^	–		
7. Self-handicapping T1	−0.07	0.03	0.19^*^	0.14	0.19^**^	0.15^*^	–	
8. Self-handicapping T2	−0.39^**^	−0.12	0.45^**^	0.31^**^	0.34^**^	0.28^**^	0.40^**^	–
Mean	28.78	29.04	34.83	35.91	12.03	13.02	34.37	33.88
SD	4.23	5.06	8.91	8.37	5.38	5.25	6.34	6.98
Skewness	0.078	0.151	−0.488	−0.807	0.874	0.546	−0.293	−0.455
Kurtosis	0.102	0.331	0.381	1.356	0.285	−0.038	−0.128	−0.389
Cronbach α	0.75	0.86	0.93	0.93	0.91	0.90	0.62	0.69
McDonald ω	0.75	0.86	0.93	0.93	0.91	0.90	0.63	0.72
Guttmann λ6	0.75	0.86	0.94	0.94	0.90	0.89	0.65	0.72

^**^p < 0.01; ^*^p < 0.05; SD, standard deviation; T1, Time 1; T2, Time 2.

### 3.2 Stability and mean-level change across waves

To make the two-wave structure of the data more explicit, rank-order stability and mean-level change were examined. As shown in Table 2, the Time 1-Time 2 correlations indicated statistically significant but modest stability for all variables. Mathematics teaching outcome expectancy showed low-to-moderate stability across the two waves, *r* = 0.29, *p* < 0.001. Anxiety related to teaching also showed low-to-moderate stability, *r* = 0.30, *p* < 0.001. Anxiety related to subject knowledge showed relatively lower stability, *r* = 0.23, *p* = 0.002, whereas self-handicapping showed the highest stability among the study variables, *r* = 0.40, *p* < 0.001. Paired-samples *t*-tests showed that mathematics teaching outcome expectancy did not significantly change from Time 1 (*M* = 28.78, SD = 4.23) to Time 2 (*M* = 29.04, SD = 5.06), *t*_(179)_ = −0.629, *p* = 0.530, *d* = 0.05. Anxiety related to teaching also did not show a significant mean-level change from Time 1 (*M* = 34.83, SD = 8.91) to Time 2 (*M* = 35.91, SD = 8.37), *t*_(179)_ = −1.413, *p* = 0.160, *d* = 0.11. Anxiety related to subject knowledge increased slightly from Time 1 (*M* = 12.03, SD = 5.38) to Time 2 (*M* = 13.02, SD = 5.25), *t*_(179)_ = −2.006, *p* = 0.046, *d* = 0.15. Self-handicapping did not significantly change from Time 1 (*M* = 34.37, SD = 6.34) to Time 2 (M = 33.88, SD = 6.98), *t*_(179)_ = −0.889, *p* = 0.375, *d* = 0.07. These findings suggest that the constructs showed statistically significant continuity across the 6-month interval, although the magnitude of the stability coefficients indicates that they were also open to individual-level change. Mean-level change was limited, with only a small increase observed in anxiety related to subject knowledge.

**Table 2:** Stability and mean-level change across Time 1 and Time 2.

Variable	Time 1 M	Time 1 SD	Time 2 M	Time 2 SD	Stability *r*	*t*	*p*	Cohen's *d*
Mathematics teaching outcome expectancy	28.78	4.23	29.04	5.06	0.29^***^	−0.629	0.530	0.05
Anxiety related to teaching	34.83	8.91	35.91	8.37	0.30^***^	−1.413	0.160	0.11
Anxiety related to subject knowledge	12.03	5.38	13.02	5.25	0.23^**^	−2.006	0.046	0.15
Self-handicapping	34.37	6.34	33.88	6.98	0.40^***^	−0.889	0.375	0.07

Stability coefficients are Pearson correlations between Time 1 and Time 2 scores. Cohen's d values are based on paired mean differences. ^**^p < 0.01. ^***^p < 0.001.

### 3.3 Two-wave structural equation model

A two-wave structural equation model was estimated to test the parallel mediating roles of anxiety related to teaching and anxiety related to subject knowledge in the association between mathematics teaching outcome expectancy at Time 1 and self-handicapping at Time 2 among preservice teachers. In the initial step, the measurement model included four latent constructs (mathematics teaching outcome expectancy, anxiety related to teaching, anxiety related to subject knowledge, and self-handicapping) and nine observed indicators. The fit statistics indicated an acceptable level of model fit: χ^2^ (21, *N* = 180) = 27.301, χ^2^/df = 1.3; CFI = 0.99; TLI = 0.99; NFI = 0.96; GFI = 0.97; IFI = 0.99; SRMR = 0.04; and RMSEA = 0.04. Taken together, these indices suggested that the measurement model was well-aligned with the data.

**Figure 1:**
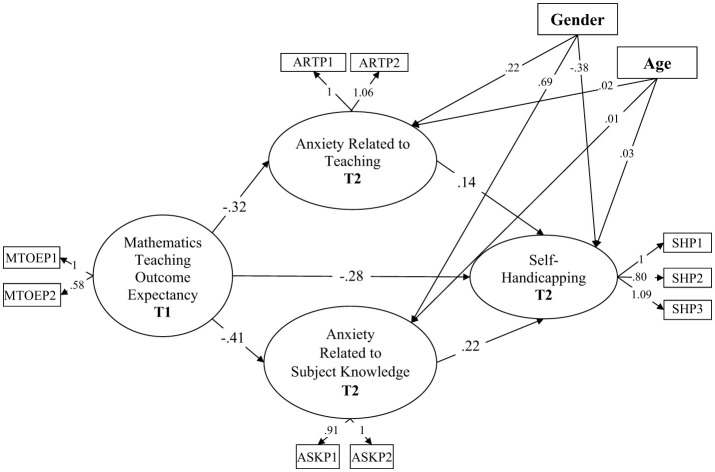
Structural equation modeling for the two-wave parallel mediation model. MTOEP, parcel of mathematics teaching outcome expectancy; ARTP, parcel of anxiety related to teaching; ASKP, parcel of anxiety related to subject knowledge; SHP, parcel of self-handicapping.

Following the evaluation of the measurement model, the structural model was analyzed to test the study hypotheses. This model was specified to examine the parallel mediating effects of anxiety related to teaching at T2 and anxiety related to subject knowledge at T2 in the link between mathematics teaching outcome expectancy at T1 and self-handicapping at T2. The fit indices again indicated acceptable fit between the model and the data: χ^2^ (35, *N* = 180) = 36.070, χ^2^/df = 1.03; CFI = 0.98; TLI = 0.99; NFI = 0.95; GFI = 0.96; IFI = 0.98; SRMR = 0.07; and RMSEA = 0.01. Age and gender were entered into the model as covariates, and their inclusion did not substantially change the overall pattern of associations. The regression estimates indicated that mathematics teaching outcome expectancy significantly and negatively predicted self-handicapping (coefficients = −0.275; *p* < 0.001), anxiety related to teaching (coefficients = −0.321; *p* < 0.05), and anxiety related to subject knowledge (coefficients = −0.407; *p* < 0.001), as presented in Figure 1.

The mediating roles in the model were examined by means of bootstrap analysis. Confidence intervals were generated using 5,000 bootstrap samples. The standardized coefficients for the indirect effects are presented in Table 3. The total effect of mathematics teaching outcome expectancy at Time 1 on self-handicapping at Time 2 was significant and negative (total effect, β = −0.409, *p* < 0.001), which supported H1. The path estimates further indicated that both anxiety related to teaching and anxiety related to subject knowledge significantly predicted self-handicapping. After anxiety related to teaching and anxiety related to subject knowledge were entered into the model, the direct effect of mathematics teaching outcome expectancy on self-handicapping remained statistically significant (direct effect, β = −0.275, *p* < 0.001).

**Table 3:** Direct, indirect, and total effects in the two-wave mediation model.

Effect	Path	β	95% CI		*p*-value
			Lower	Upper	
Direct	Mathematics teaching outcome expectancy ⇒ Anxiety related to teaching	−0.321	−0.739	−0.013	0.039
	Mathematics teaching outcome expectancy ⇒ Anxiety related to subject knowledge	−0.407	−0.679	−0.190	0.001
	Anxiety related to teaching ⇒ Self-handicapping	0.143	0.032	0.255	0.007
	Anxiety related to subject knowledge ⇒ Self-handicapping	0.216	0.070	0.425	0.001
	Mathematics teaching outcome expectancy ⇒ Self-handicapping	−0.275	−0.531	−0.088	0.001
Indirect	Mathematics teaching outcome expectancy ⇒ Anxiety related to teaching ⇒ Self-handicapping	−0.046	−0.138	−0.004	0.020
	Mathematics teaching outcome expectancy ⇒ Anxiety related to subject knowledge ⇒ Self-handicapping	−0.088	−0.219	−0.026	0.001
Total	Mathematics teaching outcome expectancy ⇒ Self-handicapping	−0.409	−0.688	−0.175	< 0.001

The indirect pathways were also significant. Mathematics teaching outcome expectancy at Time 1 had a significant indirect effect on self-handicapping at Time 2 through anxiety related to teaching at Time 2 (indirect effect = −0.046, 95% CI = [−0.138, −0.004]), supporting H2. A second significant indirect effect was observed through anxiety related to subject knowledge at Time 2 (indirect effect = −0.088, 95% CI = [−0.219, −0.026]), supporting H3. In addition, mathematics teaching outcome expectancy at Time 1 exerted a significant indirect effect on self-handicapping at Time 2 through the joint contribution of anxiety related to teaching at Time 2 and anxiety related to subject knowledge at Time 2 (parallel indirect effect = −0.134, 95% CI = [−0.289, −0.053]), supporting H4. Full details concerning the indirect pathways are reported in Table 3.

## 4 Discussion

The present study examined a two-wave mediation model in which anxiety related to teaching and anxiety related to subject knowledge were tested as parallel mediators in the relationship between mathematics teaching outcome expectancy and self-handicapping among preservice classroom teachers. The results indicated that mathematics teaching outcome expectancy was negatively associated with self-handicapping. In other words, stronger beliefs in the likely instructional effects of teaching were associated with lower levels of later self-protective behavior. Mathematics teaching outcome expectancy was also negatively associated with anxiety related to teaching and anxiety related to subject knowledge. Both anxiety dimensions, in turn, were positively associated with self-handicapping. This pattern suggests that preservice classroom teachers who are less convinced that effective teaching can improve student learning may experience greater anxiety about lesson enactment and mathematical adequacy, and that both forms of anxiety may increase the likelihood of defensive coping. The direct path from mathematics teaching outcome expectancy to self-handicapping remained significant after the mediators were included, which indicates partial mediation. To the best of the author knowledge, the present study is among the few studies to examine self-handicapping in relation to mathematics teaching outcome expectancy through two parallel anxiety pathways in preservice classroom teachers. The findings extend the existing literature by indicating that beliefs about the instructional impact of teaching may be associated with later self-protective tendencies both directly and indirectly through two distinct dimensions of mathematics teaching anxiety. This contribution is important because it provides a more differentiated account of how professional beliefs and emotional experiences may jointly shape maladaptive coping during teacher training.

Result indicates that stronger beliefs in the instructional value of teaching may reduce the tendency to rely on defensive responses during teacher preparation. Bandura (1997) emphasized that people's expectations about the likely outcomes of their actions are important for motivation and engagement. From this perspective, preservice teachers who believe that effective mathematics teaching can improve students' learning may be less likely to protect themselves through reduced effort, avoidance, or excuse-making when instructional tasks become demanding. Covington (1984) argued that self-protective responses become more likely when competence is felt to be at risk. In this respect, the present finding is theoretically understandable. It is also consistent with the studies of Urdan and Midgley (2001), who conceptualized self-handicapping as a strategy used to protect self-worth in evaluative settings. Goodarznaseri et al. (2026) similarly described self-handicapping as part of a maladaptive motivational pattern associated with self-image protection and reduced academic engagement. Within the context of mathematics teacher preparation, beliefs about the likely effects of teaching may function as a protective professional resource against later defensive coping.

The findings also indicated that anxiety related to teaching and anxiety related to subject knowledge mediated the association between mathematics teaching outcome expectancy and self-handicapping. This result is compatible with control-value theory, which proposes that anxiety becomes more likely when achievement situations are appraised as important but difficult to control (Pekrun, 2006, 2024). Mathematics teaching frequently has this structure during teacher training because it requires public explanation, instructional judgment, and visible demonstrations of competence. Previous research has often examined anxiety and beliefs about teaching mathematics together. Swars et al. (2006) and Gresham (2008) reported negative associations between mathematics anxiety and mathematics teaching efficacy in preservice teachers. Uysal and Dede (2016) reached a similar conclusion in a Turkish sample. (Abuan and Taganap 2025) reported that mathematics anxiety significantly predicted mathematics teaching anxiety in preservice teachers, whereas Lavidas et al. (2023) indicated that preservice teachers' mathematics anxiety was associated with their beliefs about and attitudes toward teaching mathematics. Given that these studies often used broader efficacy-related constructs, they should be interpreted as convergent rather than direct evidence for mathematics teaching outcome expectancy, defined here as the belief that effective teaching can influence students‘ mathematics learning. The present findings extend this convergent pattern by indicating that anxiety may help explain how self-handicapping is linked with outcome expectancy specifically, not efficacy beliefs in general.

The results suggest that the association between outcome expectancy and later self-handicapping is not carried by a single form of teaching anxiety. The results suggest that the association between outcome expectancy and later self-handicapping is not carried by a single form of teaching anxiety. Both anxiety related to teaching and anxiety related to subject knowledge appear relevant. This distinction is meaningful for classroom teacher training. Anxiety related to teaching reflects tension surrounding explanation, classroom interaction, lesson management, and assessment. Anxiety related to subject knowledge reflects concern about the adequacy of one's mathematical understanding during instruction. (Ball et al. 2008) suggest that teaching mathematics requires forms of knowledge that go beyond routine performance, and this helps explain why subject-knowledge-related anxiety may become salient during teacher training. Research by (Bjerke and Solomon 2020) and (Bjerke and Xenofontos 2024) also highlighted the developmental character of self-efficacy in teaching mathematics and the importance of subject knowledge in that process. The present findings indicate that these two anxiety dimensions should be treated as related but distinct pathways when explaining later self-protective responses.

A further finding of the present study was that anxiety related to teaching and anxiety related to subject knowledge jointly accounted for the association between mathematics teaching outcome expectancy and self-handicapping within a parallel mediation model. This result does not suggest that one anxiety dimension leads to the other. Rather, it indicates that both dimensions operate concurrently as explanatory pathways linking earlier professional beliefs with later defensive coping. Prior work suggests that beliefs related to mathematics teaching may change during teacher education and across early professional transitions (Işiksal-Bostan, 2016). The present study does not assume that mathematics teaching outcome expectancy is fully stable, and the stability analyses support this. Mathematics teaching outcome expectancy showed statistically significant but modest stability across the two waves. This indicates some continuity across the 6-month interval, but it should not be treated as a fixed personal attribute. Related research has likewise documented that efficacy in teaching mathematics develops through preparation experiences and field-based opportunities (Bjerke and Xenofontos, 2024; Charalambous et al., 2008). In this respect, the present study contributes to literature by indicating that outcome expectancy may be associated not only with teaching-related feelings but also with later behavioral tendencies that may interfere with professional learning. Studies examining self-handicapping through two parallel mathematics teaching anxiety pathways in preservice classroom teachers remain limited. This gives the present findings particular value for the literature on mathematics teacher training.

The persistence of the direct path from mathematics teaching outcome expectancy to self-handicapping indicates that mathematics teaching anxiety explained part of the association, but not all of it. Barkela et al. (2024) reported that student teachers facing self-worth threat may engage in self-protective behaviors that interfere with professional learning. This suggests that fear of negative evaluation, self-doubt, or other maladaptive coping tendencies may also contribute to the remaining direct association. Therefore, the present findings identify one meaningful explanatory route, while also indicating that the relationship between professional beliefs and self-handicapping is likely to be more complex. The weak concurrent associations involving Time 2 mathematics teaching outcome expectancy point to a further layer of this complexity. Time 2 outcome expectancy showed no meaningful association with Time 2 anxiety related to teaching or with self-handicapping. It showed only a small association with Time 2 anxiety related to subject knowledge. This pattern indicates that outcome expectancy does not function as a simple concurrent protective factor. One interpretation is that earlier outcome expectancy reflects an initial belief orientation linked to later emotional and self-protective responses. Concurrent outcome expectancy, by contrast, may itself be reshaped by intervening coursework, practicum experiences, feedback, and shifts in perceived instructional control during teacher education. The present findings should therefore be read as evidence of two-wave associations originating from earlier outcome expectancy, rather than as evidence of a stable or causal developmental process.

The findings also have practical implications for teacher education. Mathematics teaching anxiety may require more than general emotional support. It appears to operate through two related but distinct domains, and these domains point to different areas of need. Experiences centered on explanation, representation choice, classroom interaction, and error interpretation may be more relevant for anxiety related to teaching. Experiences that strengthen conceptual understanding and connect subject knowledge with instructional use may be more relevant for anxiety related to subject knowledge. Research on mathematics teacher efficacy has emphasized the importance of preparation experiences, transition points, and mastery-related opportunities in shaping professional confidence (Bjerke and Xenofontos, 2024; Işiksal-Bostan, 2016). Charalambous et al. (2008) similarly reported that fieldwork plays an important role in developing preservice teachers' efficacy beliefs in teaching mathematics. Experiences that strengthen confidence in the instructional impact of teaching may additionally help reduce vulnerability to later self-handicapping. Support directed at mathematics teaching outcome expectancy may work alongside coursework and practicum opportunities that target each anxiety domain. Together, these forms of support may help reduce self-handicapping by lowering both forms of mathematics teaching anxiety. Future research may examine how such preparation experiences shape mathematics teaching outcome expectancy and mathematics teaching anxiety over time.

## 5 Conclusions

The present study indicated that preservice classroom teachers with stronger mathematics teaching outcome expectancy reported lower levels of later self-handicapping. Anxiety related to teaching and anxiety related to subject knowledge also mediated this relationship in parallel. When these two mediators were included, the direct effect of mathematics teaching outcome expectancy on self-handicapping decreased but remained significant. This pattern suggests partial parallel mediation.

The findings extend the literature in several respects. The study focused specifically on mathematics teaching outcome expectancy instead of treating mathematics teaching efficacy as a single undifferentiated construct. This distinction is important because outcome expectancy concerns beliefs about the effects of teaching, whereas personal teaching efficacy concerns beliefs about one's own teaching capability. It also distinguished between two dimensions of mathematics teaching anxiety and linked them to self-handicapping, a variable that has received limited attention in mathematics teacher preparation. In this respect, the findings suggest that stronger beliefs in the instructional value of teaching may protect preservice classroom teachers from later defensive coping, partly by reducing anxiety related to teaching and anxiety related to subject knowledge. The results also indicate that mathematically demanding teaching situations are shaped by both professional beliefs and emotional experiences during teacher preparation.

## 6 Limitations and directions for future research

Several limitations should be considered when interpreting the findings. The sample consisted of preservice classroom teachers enrolled at a public university in Türkiye and was obtained through convenience sampling. The participants were also predominantly female. These characteristics limit the generalizability of the findings to other teacher education contexts, age groups, and cultural settings. Research with more diverse samples, different institutional contexts, and broader geographical representation would help clarify the robustness of the observed pattern.

The study used a two-wave design with a 6-month interval. This design provides a stronger basis for interpretation than a cross-sectional study, yet it does not permit strong conclusions about temporal ordering among all variables because the mediators and the outcome were assessed at the same wave. Cole and Maxwell (2003) noted that longitudinal mediation models require careful attention to temporal structure, and Maxwell and Cole (2007) showed that mediation estimates may be biased when temporal ordering is underspecified. For this reason, the present findings are better interpreted as prospective associations rather than evidence of sequential causal relations. The study also did not estimate a full cross-lagged panel model because the central aim was not to examine reciprocal temporal effects among all constructs. It was also consistent with recent methodological cautions regarding the use of CLPMs with two-wave data. Lucas (2023) noted that standard CLPMs may confound cross-lagged paths with stable trait-like associations and other unmodeled person-level sources of variance. He also argued that two-wave designs provide limited information for distinguishing among longitudinal models. Therefore, estimating a CLPM in the present study would not necessarily have provided a more defensible test of reciprocal processes. The present model is better described as a half-longitudinal mediation design, in which the predictor was measured at Time 1, and the mediators and dependent variable were measured at Time 2. Similar two-wave or half-longitudinal mediation structures have been used in recent educational and psychological research when the aim was to examine theory-driven indirect pathways rather than fully reciprocal longitudinal processes (Cengiz and Peker, 2024; Ma et al., 2024; Shek et al., 2024). Accordingly, alternative temporal pathways cannot be ruled out. For example, mathematics teaching anxiety or self-handicapping may also shape later outcome expectancy. Future studies may strengthen the design by using three or more waves and by comparing alternative longitudinal models, including cross-lagged and random-intercept models.

Another limitation is about measurement. All variables were assessed through self-report instruments. This may have increased shared method variance and may also have introduced social desirability or self-perception bias. Podsakoff et al. (2003) and Podsakoff et al. (2012) both emphasized that associations among conceptually related variables may be inflated when they are derived from the same source. Future studies would benefit from combining self-report data with classroom observations, practicum evaluations, interviews, peer reports, or instructor ratings. Mixed-method designs may be especially useful because they can clarify how self-protective responses emerge in concrete instructional settings.

A further issue relates to the measurement of self-handicapping. Reliability estimates were satisfactory for most study variables, whereas the self-handicapping measure yielded comparatively lower internal consistency values. Although the scale performed adequately enough to remain in the model, future research may benefit from alternative instruments or more context-specific assessments of self-handicapping in teacher education samples. More precise measurement may be particularly valuable in longitudinal mediation studies, where the interpretation of indirect effects depends on clear construct representation.

## Data Availability

The raw data supporting the conclusions of this article will be made available by the authors, without undue reservation.
